# Effects of Acute Bleeding Followed by Hydroxyethyl Starch 130/0.4 or a Crystalloid on Propofol Concentrations, Cerebral Oxygenation, and Electroencephalographic and Haemodynamic Variables in Pigs

**DOI:** 10.1155/2014/710394

**Published:** 2014-05-19

**Authors:** Aura Silva, Ana Liza Ortiz, Carlos Venâncio, Almir P. Souza, Luísa Maria Ferreira, Paula Sério Branco, Paula Guedes de Pinho, Pedro Amorim, David A. Ferreira

**Affiliations:** ^1^Toxicology Department, REQUIMTE, Faculdade de Farmacia, Universidade do Porto, Rua de Jorge Viterbo Ferreira, No. 228, 4050-313 Porto, Portugal; ^2^Universidad de Leon, Avenida Facultad de Veterinaria, No. 25, 24004 León, Spain; ^3^Centre for the Research and Technology of Agro-Environmental and Biological Sciences (CITAB), Animal Science Department, University of Tras-os-Montes and Alto Douro, Quinta de Prados, 5000-801 Vila Real, Portugal; ^4^Unidade Academica de Medicina Veterinária, Universidade Federal de Campina Grande, Patos, Rua Sinfrônio Nazaré, 1 Centro, 58800-240 Sousa, PB, Brazil; ^5^Chemistry Department, Faculdade de Ciencias e Tecnologia, Universidade Nova de Lisboa, EQUIMTE/CBQF, Quinta da Torre, 2829-516 Caparica, Portugal; ^6^Anaesthesiology Department, Hospital Geral de Santo Antonio, 4099-001 Porto, Portugal; ^7^Veterinary Sciences Research Center (CICV), Faculdade de Medicina Veterinaria, Universidade Lusófona de Humanidades e Tecnologias, Campo Grande 376, 1749-024 Lisboa, Portugal

## Abstract

Bleeding changes the haemodynamics, compromising organ perfusion. In this study, the effects of bleeding followed by replacement with hydroxyethyl starch 130/0.4 (HES) or lactated Ringer's (LR) on cerebral oxygenation and electroencephalogram-derived parameters were investigated. Twelve young pigs under propofol-remifentanil anaesthesia were bled 30 mL/kg and, after a 20-minute waiting period, volume replacement was performed with HES (G_HES_; *N* = 6) or LR (G_RL_; *N* = 6). Bleeding caused a decrease of more than 50% in mean arterial pressure (*P* < 0.01) and a decrease in cerebral oximetry (*P* = 0.039), bispectral index, and electroencephalogram total power (*P* = 0.04 and *P* < 0.01, resp.), while propofol plasma concentrations increased (*P* < 0.01). Both solutions restored the haemodynamics and cerebral oxygenation similarly and were accompanied by an increase in electroencephalogram total power. No differences between groups were found. However, one hour after the end of the volume replacement, the cardiac output (*P* = 0.03) and the cerebral oxygenation (*P* = 0.008) decreased in the G_LR_ and were significantly lower than in G_HES_ (*P* = 0.02). Volume replacement with HES 130/0.4 was capable of maintaining the cardiac output and cerebral oxygenation during a longer period than LR and caused a decrease in the propofol plasma concentrations.

## 1. Introduction

The choice of fluid administration in clinical situations where it is urgent to restore macrohaemodynamic function should also assure the best beneficial effects on microcirculation and tissue oxygenation [1]. Rapid restitution of intravascular volume is essential to maintain the vital organs' perfusion. There are different intravascular volume replacement regimens for providing haemodynamic stability after blood loss, including blood and its components, synthetic colloids as dextrans, gelatines, and hydroxyethyl starch (HES), or crystalloids as lactated Ringer's solution (LR) [2, 3].

HES 130/0.4 is the most recent hydroxyethyl starch which was created with a lower molecular weight and lower degree of substitution to overcome the side effects of previous heavier starches [4]. However, in June 2013 [5] the marketing authorizations for all HES products for human patients were suspended in the United Kingdom by the European Medicines Agency. This decision was based on three large randomized controlled trials [1, 6, 7] in which the use of HES was associated with higher rates of acute kidney failure or dialysis. Because HES is often used in veterinary patients, there is a concern about the implications that the drug may have in these patients. Thus, it is important to understand the advantages and drawbacks of resuscitation with HES compared to crystalloids.

Electroencephalogram-derived parameters such as the bispectral index (BIS) have been suggested to reflect changes in the cerebral perfusion during hemorrhagic shock followed by resuscitation in pigs [8] and it would be interesting to compare the effects of HES and crystalloids in this parameter. However, recent data suggest that these BIS variations are merely due to the anaesthetic depth until development of lethal hypotension at which brain electrical activity cannot be sustained [9, 10]. Near-infrared spectroscopy (NIRS) may make it possible to study the regional cerebral blood flow [11], while brain venous oxygenation (SvjO_2_) monitoring may yield useful information of general cerebral perfusion [12].

Acute bleeding and fluid replacement may also change the pharmacokinetics of intravenous drugs [13, 14] such as propofol, which is increasingly used in veterinary patients, and it is important to understand its pharmacokinetics when facing acute blood loss followed by volume replacement using crystalloid or colloid solutions.

By understanding the posthaemorrhage effects of different volume replacement solutions on brain oxygenation, electroencephalogram, cardiovascular parameters, and propofol pharmacokinetics, it is possible to improve brain and cardiovascular function and optimise the propofol titration.

This study addresses the effects of volume replacement with a crystalloid (lactated Ringer's) or a colloid (hydroxyethyl starch 130/0.4) solution on propofol plasma concentrations, cerebral oxygenation, electroencephalogram-derived parameters, and haemodynamics after severe acute bleeding in pigs under propofol and remifentanil total intravenous anaesthesia.

## 2. Material and Methods

All procedures were carried out under personal and project licenses approved by the national regulatory office (Direcção Geral de Veterinária—DGV000228).

Twelve healthy three-month-old large white pigs were used in this study. The G_LR_ pigs weighed 27.2 ± 3.1 Kg and the G_HES_ weighted 26.8 ± 4.1 Kg.

The pigs were fasted overnight but were permitted free access to water. Blood samples were withdrawn from each pig at the beginning and at the end of the study for hematologic and biochemical analysis. All pigs were submitted to general anaesthesia with propofol and remifentanil during which the methodological procedures took place and were submitted to the same monitoring and bleeding procedures. These same animals were used for another study of the effects of a remifentanil bolus on the electroencephalogram, haemodynamics, and cerebral oxygenation which benefited from the same monitoring setup and the same anaesthesia but was performed before the start of the present study in the same conditions for all animals and the effect of the bolus was allowed to pass before the start of the present study.

These pigs were randomly assigned in two groups, which differed only in the type of fluid used in the volume replacement phase; six animals received hydroxyethyl starch 130/0.4 (G_HES_) (Voluven, Fresenius Kabi, Bad Homburg, Germany) and six animals received lactated Ringer's (G_LR_) (B Braun, Melsungen, Germany).

### 2.1. Anaesthesia, Monitoring, and Equipment

All pigs were premedicated with azaperone IM 4 mg kg^−1^ (Stresnil, Janssen Animal Health, Belgium) thirty minutes prior to the beginning of anaesthesia induction. After premedication, a 22G catheter was inserted in the right auricular vein for drug and fluid administration. Two three-way stopcock valves were used to connect the intravenous catheter to the maintenance lactated Ringer's (LR) delivery line and to the lines delivering propofol 1% (Fresenius Kabi, Bad Homburg, Germany) and remifentanil 20 *μ*g mL^−1^ (Ultiva, GSK, Midlessex, UK). An infusion pump (Braun, Melsungen, Germany) was used for the administration of LR at a constant infusion rate of 6 mL kg^−1^ h^−1^ + 1 mL kg^−1^ h^−1^ for each kg above 20 kg of weight [15] during the entire study period. Propofol and remifentanil were delivered using two perfusion pumps (Asena GH, Alaris Medical Systems, San Diego, CA) controlled by the RugLoop II Waves software developed by Tom De Smet (Demed Engineering, Temse, Belgium) and Michel Struys (Ghent University, Gent, Belgium) running in a personal computer.

Induction of anaesthesia was performed with a propofol bolus of 4 mg kg^−1^ while pigs were breathing 100% oxygen via a facial mask. This was followed by tracheal intubation with a 6.5 mm endotracheal tube. The pigs were mechanically ventilated with a mixture of 20% air + 80% oxygen, using a Datex Carestation ventilator, with a tidal volume of 10 mL kg^−1^, respiratory rate of 12 to 14, and inspiration expiration ratio of 1 : 3, with adjustments according to the observed ETCO_2_ in order to obtain a PaCO_2_ of 40 ± 4 mmHg.

After induction of anaesthesia, a propofol constant infusion was started at a rate of 15 mg kg^−1^ h^−1^  which remained unchanged during the entire study period. Simultaneously, a remifentanil constant infusion was started at a rate of 0.3 *μ*g kg^−1^ min^−1^. After ending all monitoring procedures, the remifentanil infusion rate was decreased to 0.2 *μ*g kg^−1^ min^−1^ and was maintained unaltered during all the study period. Peripheral oxygen saturation (SpO_2_) was recorded by placing the probe on the pig's tongue, and heart rate was collected by three ECG electrodes placed according to Academy of Veterinary Cardiology Committee.

### 2.2. Haemodynamic Monitoring

After reaching stable anaesthesia (total muscle relaxation, absence of palpebral reflex, and absence of haemodynamic response to interdigital space clamping), the following invasive instrumentation procedures were performed in each pig for placement of arterial catheters in the left femoral artery (Leadercath, Vygon Corporation, PA) for monitoring continuous blood pressure and a 16 gauge catheter in the right femoral artery (Abbot Animal Health, IL) for passive bleeding. A surgical approach to the ventral cervical region was used in all pigs for introducing two 7F Swan-Ganz optic catheters (Edwards, Life Sciences, Irvine, CA): one in the internal jugular vein with its optic tip in the* sinus petrosus ventralis*, for collecting data from venous blood oxygen saturation (SvjO_2_), and the other in the pulmonary artery, via left external jugular vein, for collecting data from central venous pressure, pulmonary artery pressure and cardiac output by the thermodilution method, and pulmonary pressure.

A multiparametric haemodynamic monitor (Datex-Ohmeda S/5, Helsinki, Finland) was used to collect all haemodynamic and ventilatory data. The SvjO_2_ was measured using an Oxymetrix 3 monitor (Abbott Laboratories, North Chicago, IL, USA) also connected to the S/5 Datex monitor. All data was recorded every five seconds in a personal computer, via a RS-232 interface, running the RugLoop II Waves software.

### 2.3. Brain Monitoring

The fur on the skin over the fronto-occipital region was shaved, and the skin grasped with fine sandpaper and cleaned with acetone, and a BIS adhesive electrode (Zipprep, Aspect Medical Systems, Natick, MA) was placed in the left side of the head in the following position: number 1 electrode was placed over the external occipital protuberance; number 2 and number 4 electrodes were placed over the left hemisphere; number 3 electrode was placed over the rostral left portion of the frontal bone, at the level of the left eye. The EEG was recorded at 256 Hz using a BIS XP monitor (Aspect Medical Systems, Natick, MA). The monitor also recorded the bispectral index (BIS), electromyographic activity (EMG), signal quality index, spectral edge frequency 95% (SEF) suppression ratio (SR), and total power (TP). Data from the BIS monitor were recorded every second in the personal computer running the RugLoop II Waves software.

The near-infrared spectroscopy INVOS monitor 4100 with the software version 11.16.16 (Somanetics Corporation, Troy, MI) was used continuously to monitor changes in regional oxygen saturation (rSO_2_) in the left side of the head using its noninvasive electrodes (SomaSensors, Somanetics Corporation, Troy, MI). Data from cerebral oxygen saturation given by the INVOS was recorded continuously using ASYS software [16] running in a second personal computer, with the clock synchronized with that in the computer running the RugLoop II Waves software.

An Oxymetrix 3 monitor (Abbott Laboratories, North Chicago, LL, USA) with a 7F Swan-Ganz optic catheter (Edwards, Life Sciences, Irvine, CA), with its optic tip placed in the* sinus petrosus ventralis*, was used for collecting data from venous blood oxygen saturation (SvjO_2_), for monitoring the overall brain oxygenation.

### 2.4. Experimental Protocol

After completing all the necessary instrumentation procedures, 30 mL kg^−1^ of blood was passively removed from each pig through the right femoral artery, during approximately 20 minutes. Twenty minutes after the bleeding has stopped, the volume replacement phase started at 999 mL h^−1^ with LR or HES 130/0.4 solution, depending on the treatment group. The volume of HES 130/0.4 administered for volume replacement was 20 mL kg^−1^ [17], and the volume of Ringer solution administered was 25% higher (25 mL kg^−1^) than that used for HES based on preliminary studies. These volumes were administered in the external jugular vein, via the side port of the Swan-Ganz catheter introducer (7F Intro-Flex, I500F7C, Edwards, Life Sciences, Irvine, CA).

After the end of the volume replacement, all animals were maintained under the same rates of propofol and remifentanil for an additional one hour. At the end of the study, the pigs were euthanized under general anaesthesia with intravenous potassium chloride. At the end of the study all the pigs were subjected to necropsy to check for correct positioning of the catheters and direct visualization of the internal organs.

Cardiac output measurements using the thermodilution method were performed just before the start of bleeding (*T*_0_), when 50% of the blood volume was removed, at the end of the bleeding, at the end of the twenty-minute waiting period, at the end of the volume replacement, and at the end of the study.

Arterial blood samples were also collected for blood gas analyses right before bleeding, at the end of the waiting period, in the end of the volume replacement, and in the end of the study using a Gem Premier 3000 analyser (Instrumentation Laboratory, Massachusetts, USA).

During the study period, 3 mL arterial blood samples were collected from the right femoral artery into heparin containing tubes for propofol and propofol metabolites quantification in the plasma right before bleeding (*C*_*p*_0), 10 (*C*_*p*_1) and 15 (*C*_*p*_2) minutes after the beginning of bleeding, at the end of bleeding (*C*_*p*_3), ten minutes after the beginning of the waiting period (*C*_*p*_4) and at the end of the waiting period (*C*_*p*_5), and 15 (*C*_*p*_6), 30, 45, and 60 minutes after the beginning of volume replacement (*C*_*p*_7, *C*_*p*_8, and *C*_*p*_9). After blood collection the plasma was separated through centrifugation at 3000 rpm for 15 minutes and was immediately placed at −77°C and stored until analysis.

Propofol plasma concentrations as well as its free metabolites (2,6-diisopropyl-1,4-quinol and 2,6-diisopropyl-1,4-quinone) [18] were determined by gas chromatography mass spectrometry according to Guitton and colleagues [19] with some adjustments, as described in Silva and colleagues [20]. For the calibration curve, the nonconjugated metabolites were chemically synthesized since these compounds are not commercially available. The purified metabolites (>95%) were subsequently used as GC-MS standards.

The plasma concentrations of propofol were divided by the total volume of propofol administered to check for influences of the duration of propofol infusion on the final concentrations of the drug.

### 2.5. Electroencephalographic Analysis

The BIS monitor recorded the raw EEG at 256 Hz which was further converted to be processed offline using the MATLAB software (MathWorks, Natick, MA). The signal's sampling frequency was first decreased 2 times, resulting in 128 Hz. The indices approximate entropy (AE) and permutation entropy (PE) were derived from EEG epochs of 8 seconds after filtering using a butterworth bandpass filter of 8th order with cutoff frequencies of 0.5 and 30 Hz followed by removal of the mean value of the signal in order to get out any threshold. AE and PE were computed according to published algorithms [21–23]. Briefly, the calculation of the AE depends on three factors: the embedding dimension (*m*), the number of samples considered for each calculation (*N*), and the noise threshold (*r*). In this study *N* = 1024, *m* = 2, and *r* = 0.2 were selected for AE calculation, based on previous studies [24]. For the PE calculation, the length of subvectors (*m*) and the analyzed signal interval (length *N*) are main factors. In this study, we used *m* = 3 and *N* = 1024. A more detailed description of the AE and PE calculation can be found in published works [22, 23, 25].

The spectral parameters were corrected for the presence of burst suppression patterns, using the values of SR (suppression ratio) recorded by the BIS monitor, according to the correction factor proposed by Rampil [26]. The same correction factor was applied to PE resulting in BSPE, calculated as follows: BSPE = PE × (1 − SR/100) [20, 27].

The raw EEG was recorded along with the BIS, EMG, SEF, and TP,   all derived automatically from the BIS monitor. These parameters were included in the overall analysis of the indices of anaesthetic depth.

### 2.6. Statistics

Four different study periods were analysed: (1) the bleeding period; (2) the 20-minute waiting period; (3) the volume reposition period; and (4) the final period until euthanasia. Each period was normalized in 10 different 10% parts. Each 10% part represented the average of 30 seconds of consecutive measurements.

The percentage of oxygen extraction (SpO_2_-SvO_2_) was also analyzed.

Data were tested for normal distribution and homogeneity of variance using the Shapiro-Wilk normality test and Levene test, respectively. Haemodynamic, cerebral oxygenation, propofol and metabolites plasma concentrations, and electroencephalographic data were compared within groups and between groups using two-way repeated measures analysis of variance, with Bonferroni corrections for pairwise comparisons which was performed separately for each study period.

Correlation analysis was performed between the studied parameters using the Pearson and Spearman Rank correlation coefficients for normal and non-Gaussian data, respectively. Correlation analysis was performed during the bleeding period and during the volume replacement period.

Statistical analysis was performed using Graphpad prism (Version 5, GraphPad Software Inc., San Diego, CA). Data are expressed as mean ± SD; *P* < 0.05 was considered statistically significant.

## 3. Results

The bleeding period had a mean duration of 19.8 ± 0.8 (G_LR_) and 20.3 ± 2.6 (G_HES_), the waiting period lasted for 24.9 ± 2.7 (G_LR_) and 24 ± 3.2 (G_HES_) minutes, the volume replacement period lasted for 43.8 ± 5.7 (G_LR_) and 33.1 ± 4.8 (G_HES_) minutes, and the period between the end of replacement and the end of study was 53.5 ± 16.1 (G_LR_) and 54.1 ± 14.9 (G_HES_) minutes. The average blood volume withdrawn was 680 ± 78 mL in the G_LR_ and 671 ± 104 mL in the G_HES_.

The blood temperature of the pigs was maintained between 39 and 40°C (normothermia for pig) during the whole anaesthesia. The changes in arterial blood gases were comparable between the two groups throughout the whole study (Table 1). There were no significant changes in the oxygen extraction (SpO_2_-SvjO_2_) between the two groups, although there were variations throughout time (*P* = 0.03) (Table 2).

**Figure 1:**
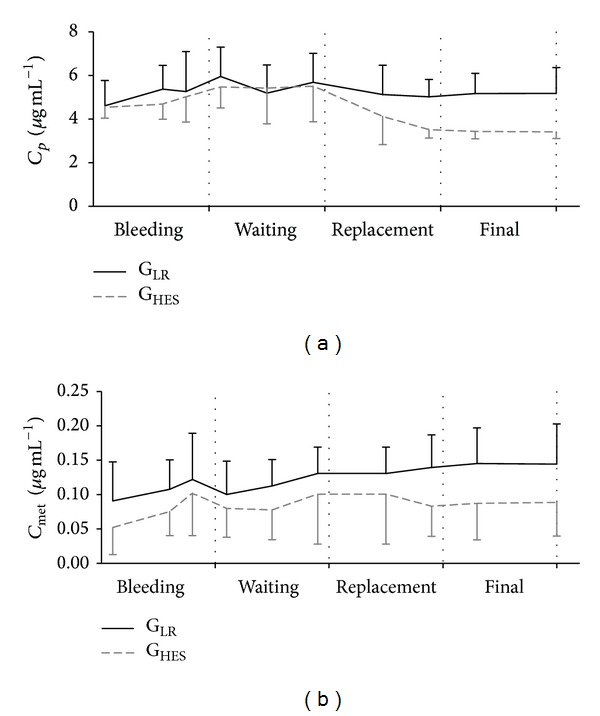
Propofol plasma concentration (*C*_*p*_—*μ*g/mL) and propofol free metabolites (*C*_met_—*μ*g/mL) during the four phases of the study. The two groups are shown: G_LR_—black line and G_HES_—dashed grey line.

*Bleeding Period*. Propofol plasma concentration (*C*_*p*_) increased during bleeding (*P* < 0.01), and propofol free metabolites (*C*_met_) also changed throughout time (*P* < 0.001), increasing until 15 minutes after the beginning of bleeding and decreasing from 15 minutes to the end of bleeding (Table 2 and Figure 1).

Regarding the EEG-derived parameters, during bleeding, BIS (*P* = 0.04) and TP (*P* < 0.001) decreased significantly. No significant changes were found in the other studied EEG-derived parameters or in any parameter between the two groups (Table 3).

**Figure 2:**
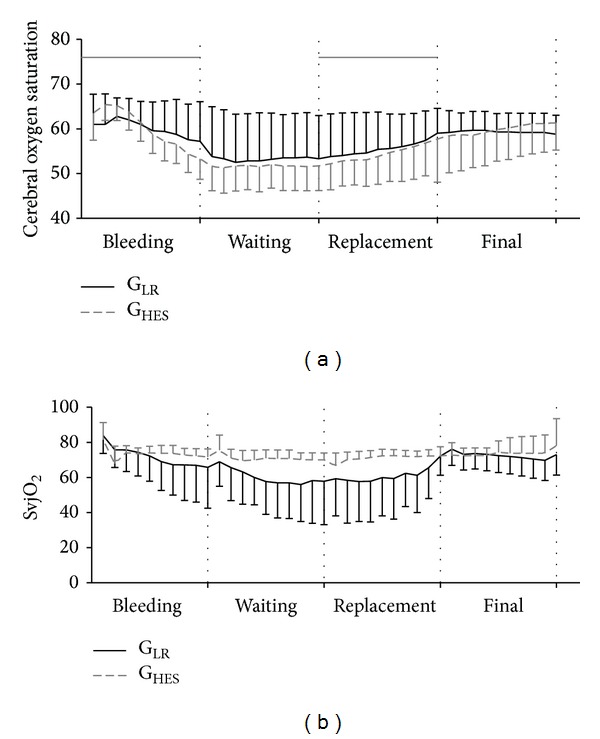
Cerebral oxygenation parameters during the four phases of the study: cerebral oxygen saturation (INVOS) (a) and SvjO_2_ (%) (b). The two groups are shown: G_LR_—black line and G_HES_—dashed grey line.

SvjO_2_ and cerebral oxygen saturation reflected by INVOS decreased throughout bleeding (*P* = 0.039), but no differences were found between the two groups (*P* > 0.1) as shown in Table 2 and Figure 2.

**Figure 3:**
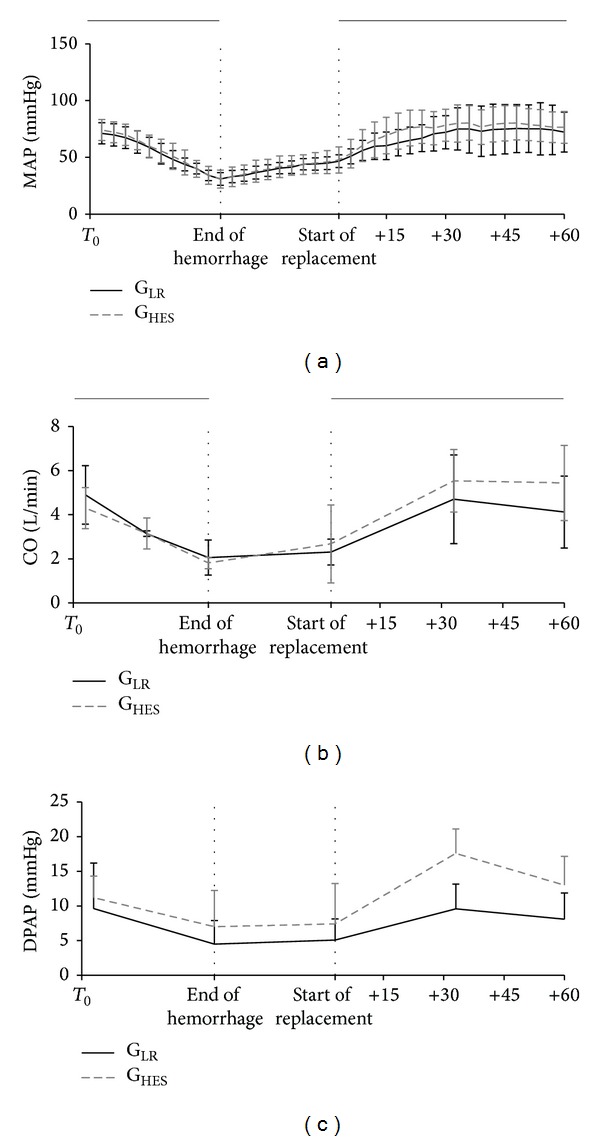
Haemodynamic variables during the four phases of the study: mean arterial pressure (MAP: mmHg), cardiac output (CO: L/min), and diastolic pulmonary artery pressure (DPAP: mmHg). The two groups are shown: G_LR_: black line and G_HES_: dashed grey line.

During the bleeding period there was a significant decrease in blood pressure (*P* < 0.001) and no significant differences were found between groups. In G_LR_, MAP decreased by 54.6% from the beginning to the end of bleeding and in G_HES_ it decreased by 52.3% in *T*_10_ when compared to baseline (*T*_0_) (Table 2 and Figure 3). There were no significant changes in HR throughout time (*P* = 0.98) or between groups (*P* = 0.52) (Table 2). CO decreased throughout bleeding (*P* < 0.001) similarly in both groups (*P* = 0.47) and DPAP (*P* < 0.05) decreased in both groups as shown in Table 2 and Figure 3.

### 3.1. Correlation Analysis

During bleeding there were significant correlations between MAP and CO (*r* = 0.70; *P* < 0.001), HR and *C*_*p*_ (*r* = −0.59; *P* < 0.001), CO and cerebral oxygen saturation (*r* = 0.54; *P* < 0.001), and CO and *C*_*p*_ (*r* = −0.55; *P* < 0.05).

Regarding the EEG-derived parameters, SR and TP correlated negatively with each other (*r* = −0.58; *P* < 0.001) and AE correlated with PE (*r* = 0.69; *P* < 0.001) and SRPE (*r* = 0.78; *P* < 0.001).

*Waiting Period*. At the end of the waiting period a significant increase from the values previous to bleeding was observed in plasma lactate levels from 2.1 ± 1.1 to 2.8 ± 1.0 in G_LR_ (*P* < 0.01) and from 2.0 ± 1.0 to 2.2 ± 0.68 in G_HES_ (*P* < 0.01).

No changes were found in *C*_*p*_ and *C*_met_ between or within groups (Table 2 and Figure 1).

Regarding the EEG-derived parameters, TP decreased during the waiting period (*P* < 0.001) and PE increased (*P* = 0.01), as shown in Table 3.

SvjO_2_ decreased (*P* < 0.05) and cerebral oxygen saturation showed no significant differences (*P* = 0.43) (Table 2 and Figure 2). There was a significant decrease in the hematocrit from 26.1 ± 4.6% to 17.1 ± 2.5% in G_LR_ (*P* < 0.01) and from 26.4 ± 5.6% to 20.9 ± 4.2% in G_HES_ (*P* < 0.01). There were no significant differences between groups in these parameters. MAP increased (*P* < 0.001) similarly in both groups. CO and HR showed no significant changes throughout time (*P* > 0.1) as shown in Table 2.

No significant differences between groups were found in this period in any parameter.

*Volume Replacement Period*. *C*_*p*_ did not change significantly in this period (Table 2 and Figure 1).

The TP of the EEG increased significantly (*P* < 0.001) and PE decreased significantly during replacement (*P* = 0.01) as shown in Table 3.

MAP (*P* < 0.001), HR and CO (*P* < 0.05), DPAP (*P* < 0.01), SvjO_2,_ and cerebral oxygen saturation (*P* < 0.01) increased significantly in both groups (Table 2 and Figure 2).

No significant differences between groups were found in this period in any parameter.

### 3.2. Correlation Analysis

During volume replacement, correlations were found between MAP and *C*_*p*_ (*r* = −0.66; *P* < 0.01), HR and CO (*r* = 0.81; *P* < 0.001), and HR and *C*_*p*_ (*r* = −0.52; *P* = 0.02).

Regarding the EEG-derived parameters, there were correlations between TP and HR (*r* = 0.62; *P* < 0.001) and a strong correlation between TP and SR (*r* = −0.70; *P* < 0.001). BIS showed correlation with SRSEF (*r* = 0.58*P* < 0.001). TP was correlated with *C*_*p*_ (−0.68; *P* < 0.001). AE was correlated with PE (*r* = 0.60; *P* < 0.001) and with SRPE (*r* = 0.76; *P* < 0.001).

*Final Phase*. At the end of the study, the *C*_*p*_ was significantly higher in the G_LR_ than the G_HES_ (*P* = 0.02), but the propofol metabolites concentration was not different between groups (*P* = 0.06). When the *C*_*p*_ values were divided by the total volume of propofol administered to check for influences of the duration of propofol infusion on the final concentrations of the drug, the values were still significantly lower in G_HES_ (*P* = 0.033; G_LR_ with 0.07 ± 0.02 versus G_LR_ with 0.05 ± 0.01). There were no significant differences in the total volume of propofol administered between groups (*P* = 1.0).

No significant differences between groups were found in this period in any of the remaining parameters.

MAP decreased significantly in the final hour (*P* = 0.02), HR showed no changes (*P* = 0.07), and CO decreased only in the G_LR_ (*P* = 0.03) but was kept constant on the G_HES_ (*P* = 0.77) (Table 2 and Figure 3). Similarly, SvjO_2_ decreased significantly in G_LR_ (*P* = 0.008) but was unaltered in G_HES_ (*P* = 0.64) (Table 2 and Figure 2). There were significant differences in DPAP between groups (*P* = 0.04).

Regarding the EEG-derived parameters, no changes were observed in any of the parameters in the final period (Table 3).

All of the pigs survived the study period. Postmortem evaluation during necropsy revealed correct positioning of all catheters and no macroscopic damage in the internal organs.

## 4. Discussion

This study had two major objectives: (1) to investigate the effects of acute bleeding on propofol plasma concentrations, cerebral oxygenation, EEG-derived parameters, and haemodynamics and (2) to compare the effects of volume replacement with a colloid (hydroxyethyl starch 130/0.4) and a crystalloid (lactated Ringer's) on the same parameters in pigs under propofol and remifentanil total intravenous anaesthesia.

The amount of blood withdrawn from each pig in our study represents around 50% of the pig's total blood volume [28], causing a severe haemodynamic depression and a decrease in the cerebral oxygenation, accompanied by an increase in the propofol plasma concentrations and electroencephalographic depression, reflected in a decrease in TP and BIS.

At the end of the volume replacement with HES or with LR, the plasma concentrations of propofol were higher in the LR group, when compared to the HES group, although no significant differences were observed in the EEG parameters between the two groups.

The increased propofol concentrations observed in our study after bleeding are in agreement with reports from previous studies that also observed an increase in plasma concentrations of propofol after hemorrhagic shock [5, 13, 15]. However, our study reveals different effects caused by volume replacement with crystalloid or colloid solutions on the pharmacokinetics of propofol. In a study in pigs fluid resuscitation was found to restore the pharmacokinetic alteration of propofol after hemorrhagic shock, but not the pharmacodynamic alteration [15]. The mechanism is unclear, but some reports suggest that it could be explained by an increase in the unbound propofol after aggressive haemodilution [29]. In another study in pigs the effect of three kinds of fluid infusion after high-volume blood loss on the pseudo-steady-state propofol concentration was compared: a volume of LR or hydroxyethyl starch equivalent to the blood withdrawn and a threefold volume of LR. The results showed that the pseudo-steady-state concentration is influenced differently depending on the method of fluid infusion after haemorrhage [30]. In the present study, differences in the circulatory blood volume between the two groups could explain the different concentrations of propofol obtained. However, it could also be explained by a possible interaction between HES and propofol, as found in previous laboratorial work [31, 32].

According to distinct characteristics of the physiologic solutions, colloids may assure better tissue perfusion when compared to crystalloids [33]. During the volume replacement phase, HES 130/0.4 could have assured a more efficient hepatic, renal, and pulmonary perfusion by increasing tissue microcirculation, which may have occurred less efficiently in the LR group [34]. However, because there were no significant differences in cardiac output between the two groups during replacement, the existence of overall supply differences between the two groups cannot be inferred.

Near-infrared spectroscopy (NIRS) such as the INVOS monitor used in this study may be used to noninvasively and continuously monitor changes in the regional oxygen saturation, reflecting the balance between cerebral oxygen supply and demand [11, 35]. In normal physiological conditions, changes in cerebral blood flow result in changes in oxygen delivery to the peripheral cortex, which could be detected by NIRS. On the other hand, the brain venous blood oxygen saturation (SvjO_2_) monitoring provides information about the global brain oxygenation but does not provide information about regional cortex blood supply [36]. Cerebral oxygen saturation recorded with the INVOS monitor has been shown to have an excellent correlation with the invasive methods of assessing cerebral oxygenation in a study in humans [11]. The decrease in cerebral oxygen saturation values reflected by INVOS during bleeding in our study followed the decrease in cardiac output and preload.

In our study, both cerebral oxygen saturation reflected by INVOS and SvjO_2_ decreased during bleeding reflecting changes in cerebral perfusion and oxygenation caused by hypovolemia. In healthy individuals, the brain blood supply is under regulation of the brain itself, if the mean arterial pressure is kept between 60 and 160 mmHg [37]. Mean arterial pressure values below 60 mmHg, such as those observed in our study, may cause a reduction in cerebral blood flow [38]. Furthermore, diastolic pulmonary pressure values which reflect the preload decreased during bleeding which may also explain the possible decrease in brain perfusion.

It is known that hypovolemia increases the effect of propofol in the brain [13, 39] which has been explained mainly by an increase in drug concentration induced by a reduction in the distribution volume and clearance and by an increase in end-organ sensitivity. Recent reports suggest that increases in the unbound propofol during shock could explain this mechanism [29]. This could have been the origin of the EEG changes observed during bleeding in the present study. On the other side, some studies also show the potential of the EEG to reflect ischemia during surgery [40–43]. The burst suppression seems to reflect cerebral ischemia due to the decrease in the amplitude of the EEG normally associated with cerebral ischemia. However, because in the present study bleeding also caused an increase in the propofol plasma concentrations, it is difficult to tell if the increase observed in SR and TP was due to hypotension or a relative increase in the propofol concentrations.

Despite the significant decrease in MAP caused by bleeding, no compensatory increase in heart rate was observed. This might be related to the marked resetting effect of propofol on the reflex set point previously observed in humans [44]. During the 20-minute waiting period after bleeding, animals revealed a physiologic capacity to minimize the haemodynamic depression. The endogenous homeostatic vasopressor mechanisms, including the sympathetic nervous system, the renin-angiotensin-aldosterone system, and local determinants of vascular tone, including nitric oxide and endothelin, are responsible for the initial compensatory mechanisms for hypovolemia. Once the compensatory reserve of these homeostatic mechanisms is exceeded, cardiovascular decompensation and shock may occur [45].

During the 20-minute waiting period, there were no changes in the propofol plasma concentrations, and the decrease in SvjO_2_ and in the electroencephalographic depression reflected by TP was more evident. Although in our study it was not possible to observe significant changes in any of the other EEG-derived parameters, this observation supports the hypothesis that EEG-derived parameters may reflect changes in brain hypoperfusion [40, 46, 47].

Fluid replacement caused a recovery in the haemodynamic variables and in cerebral oxygenation both in the group that received LR and in the group that received the HES. There were also changes in the EEG parameters with an increase in the TP, but with no differences between the two groups during the volume replacement phase. However, approximately one hour after the end of the volume replacement and when comparing with the end of volume replacement values, the group that received LR showed a decrease in cardiac output values and lower SvjO_2_ values and in cerebral oxygen saturation reflected by INVOS. This did not occur with the group that received HES that showed similar CO and SvjO_2_ values when compared to the end of volume replacement. This difference might be related to a better preload maintenance with HES as can be observed in the diastolic pulmonary pressure values.

The SvjO_2_ is an invasive technique to measure cerebral oxygenation using the internal jugular venous blood oxygen saturation. However, its use is not straightforward and difficulties in its use may be related to potential malposition of catheter, resulting in contamination with extracerebral blood, as well as motion artefacts, which may render readings obtained by SvjO_2_ unreliable [12, 48].

Furthermore, it has been shown that in pigs the cerebral outflow is not only via the internal jugular vein [49]. This may explain the almost absent response of SvjO_2_ to bleeding in one of the groups in our study. In our study, the reversal of the haemodynamic depressing effects of bleeding was similar in both volume replacement regimens, which is in agreement with the observations of a recent study in human patients with septic shock [50]. Nevertheless, the maintenance of a higher CO and cerebral oxygen saturation reflected by INVOS reveals a better capacity of HES to maintain the intravascular volume and cerebral perfusion after replacement, with a longer lasting effect than LR. These results are in agreement with previous suggestions that colloids improve cardiac performance in patients with hypovolemia [51, 52] and are probably related to the fact that crystalloids are electrolyte solutions that lack intrinsic colloid osmotic pressure, entering the interstitial space [53] and remaining in less of 30% of the total volume administered in the intravascular space [51, 54]. On the other hand, colloids have an osmotic pressure similar to plasma, staying largely confined to the intravascular space; this allows an increase in mean arterial pressure with greater longevity in cases of severe hypovolemia [55].

Some limitations of this study should be noted. The BIS as a monitor of anaesthetic depth in animals has not been validated. Although several studies showed that BIS decreased with increasing anaesthetic doses [56–58]; the index was developed from a database of electroencephalograms recorded in humans and caution is required when interpreting its values in animals. Another important point is the variation in the duration of study periods, as they might have introduced variability in the results.

Also, because an isobaric hemorrhage model was not adopted in this study, different metabolic states could be observed in the studied groups after bleeding which could compromise the comparisons performed. However, both groups of animals underwent similar changes in mean arterial pressure values during bleeding and also similar blood parameters values indicating similar metabolic state. Another limitation may be related to the fluid infusion schemes adopted. The fluid infusion was chosen based on preliminary trials in which a similar haemodynamic recovery (considering MAP values) was observed. However, after analysis of the data in the present paper, namely, the cardiac output and pulmonary artery diastolic pressure, it is possible to conclude that, although the recovery during replacement was similar in both groups, the preload seemed to be better maintained by HES when the fluids were no longer being administered.

## 5. Conclusions

In conclusion, after around 50% of the total blood, the intravenous delivery of HES 130/0.4 was associated with a decrease in propofol plasma concentrations indicating a possible effect of HES in the propofol pharmacokinetics. Volume replacement with lactated Ringer's and HES 130/0.4 had similar restoration of all the physiologic parameters, but HES 130/0.4 was capable of maintaining the cardiac output and cerebral oxygenation until one hour after the end of its administration, while lactated Ringer's was not. The INVOS monitor seems promising for cerebral oxygenation monitoring in veterinary anaesthesia.

## Figures and Tables

**Table 1:** Results from arterial blood gas analysis performed before the beginning of bleeding (1), at the end of the waiting period (2), in the end of the volume replacement (3), and in the end of the study one hour after the end of the volume replacement (4).

	Before bleeding		End of waiting		End of volume replacement		End of study	
	G_LR_	G_HES_	G_LR_	G_HES_	G_LR_	G_HES_	G_LR_	G_HES_
PH	7.45 ± 0.06	7.44 ± 0.03	7.4 ± 0.08	7.4 ± 0.08	7.5 ± 0.07*	7.5 ± 0.05*	7.5 ± 0.06	7.46 ± 0.04
PCO_2_ (mmHg)	46.2 ± 5.8	47.5 ± 4.1	48.5 ± 6.9	48.9 ± 9.1	44.3 ± 6.4*	43.0 ± 3.9*	44.8 ± 5.6	45.9 ± 3.5
PO_2_ (mmHg)	217.4 ± 73.9	188 ± 48.8	230.5 ± 62.0	195.5 ± 38.0	233.5 ± 49.9	221.0 ± 65.0	225.0 ± 40.9	201.8 ± 50.6
NA^+^ (mEq/L)	134.1 ± 5.1	136 ± 2.0	133.5 ± 4.8	137.4 ± 2.2	133.7 ± 5.0	137.1 ± 1.3	134.2 ± 5.0	137.9 ± 1.64
K^+^ (mEq/L)	5.8 ± 1.7	5.1 ± 1.2	5.3 ± 1.0	4.6 ± 0.73	5.13 ± 1.0	4.0 ± 0.5	4.9 ± 1.2	4.40 ± 0.52
CA^++^ (mg/dL)	1.1 ± 0.22	1.2 ± 0.2	1.1 ± 0.1	1.1 ± 0.10	1.1 ± 0.04	0.98 ± 0.21	1.2 ± 0.3	1.18 ± 0.22
GLUC (mg/dL)	60.9 ± 22.6	57.9 ± 13.5	63.3 ± 22.6	80.2 ± 25.7**	60.3 ± 28.2	81.5 ± 15.9	68.6 ± 27.8	68.3 ± 13.3
LACT (mmol/L)	2.1 ± 1.1	2.0 ± 1.0	2.8 ± 1.0*	2.2 ± 0.68*	2.9 ± 0.7^*£*^	1.7 ± 0.5^*£*^	1.8 ± 1.0^∗∗*£*^	1.11 ± 0.34^∗∗*£*^
HEMATOC (%)	26.1 ± 4.6	26.4 ± 5.6	17.1 ± 2.5**	20.9 ± 4.2**	15.9 ± 1.13	14.5 ± 2.1	18.2 ± 4.2	17.5 ± 3.5
HCO_3_^−^ (mEq/L)	31.6 ± 2.5	32.4 ± 2.0	31.0 ± 1.6**	30.9 ± 0.9**	30.5 ± 1.4	30.0 ± 2.73	31.6 ± 1.1*	32.6 ± 1.39*
PT (g/dL)	4.3 ± 0.8	4.1 ± 0.25	—	—	—	—	3.2 ± 0.6^#^	2.78 ± 0.19^#^

G_LR_: group that received lactated Ringer's for volume replacement; G_HES_: group that received hydroxyethyl starch for volume replacement. Mean ± standard deviation is shown. Significantly different from the previous measurement: *P < 0.05, **P < 0.01 level. ^£^Significant differences between groups at the *P* < 0.05 level. ^#^Significant differences between the measurement “before bleeding” and the measurement “end of study.”

**Table 2:** Mean arterial pressure (MAP: mmHg), heart rate (HR: bpm), cardiac output (CO: L min^−1^), SvjO_2_ (%), cerebral oxygen saturation given by the INVOS, % of oxygen extraction (SpO_2_-SvjO_2_), propofol plasma concentration (*C*_*p*_: *μ*g/mL), and propofol metabolites concentration (*C*_met_: *μ*g/mL) at each study phase: A: right before the start of bleeding; B: at the end of bleeding/start of the waiting period; C: at the end of the waiting period/start of the volume replacement period; D: at the end of the volume replacement period/start of the final phase; and E: at the end of the study. G_LR_: group that received lactated Ringer's for volume replacement; G_HES_: group that received hydroxyethyl starch for volume replacement. Mean ± standard deviation is shown. The number of animals (*N*) in each group for each parameter is shown.

	MAP (mmHg)		HR (bpm)		CO (L min^−1^)		SvjO_2_ (%)		Cerebral oxygen saturation		% of oxygen extraction (SpO_2_-SvjO_2_)		*C*_*p*_ (*μ*g/mL)		*C*_met_ (*μ*g/mL)	
	G_LR_ (*N* = 6)	G_HES_ (*N* = 6)	G_LR_ (*N* = 6)	G_HES_ (*N* = 6)	G_LR_ (*N* = 6)	G_HES_ (*N* = 6)	G_LR_ (*N* = 6)	G_HES_ (*N* = 6)	G_LR_ (*N* = 6)	G_HES_ (*N* = 6)	G_LR_ (*N* = 6)	G_HES_ (*N* = 6)	G_LR_ (*N* = 5)^¤^	G_HES_ (*N* = 5)^¤^	G_LR_ (*N* = 5)^¤^	G_HES_ (*N* = 5)^¤^
A	72.1 ± 11.3	73.3 ± 6.9	76.2 ± 16.2	76.3 ± 17.5	4.9 ± 1.3	4.3 ± 0.9	83.8 ± 10.1	81.5 ± 9.8	61.0 ± 6.8	63.5 ± 6.0	14.6 ± 6.4	23.4 ± 3.4	4.6 ± 1.2	4.5 ± 0.5	0.09 ± 0.06	0.05 ± 0.04
B	33.7 ± 5.8**	34.9 ± 6.14**	77.1 ± 24.7	85.8 ± 28.2	2.0 ± 0.8**	1.8 ± 0.2**	68.9 ± 13.9**	75.6 ± 8.5**	53.8 ± 11.1**	51.5 ± 5.3**	30.3 ± 15.4	29.7 ± 4.9	6.0 ± 1.3**	5.5 ± 0.9**	0.10 ± 0.05	0.08 ± 0.04
C	47.4 ± 6.4**	51.3 ± 10.6**	77.9 ± 28.4	80.9 ± 34.3	2.3 ± 0.6	2.7 ± 1.8	59.3 ± 21.1*	66.5 ± 7.6*	53.8 ± 9.5	52.2 ± 5.8	35.9 ± 22.2	30 ± 4.3	5.7 ± 1.3	5.5 ± 1.6	0.13 ± 0.04	0.10 ± 0.07
D	79.7 ± 21.6**	82.8 ± 17.7**	81.9 ± 19.2*	90.1 ± 20.9*	4.7 ± 2.0*	5.5 ± 1.4*	76.1 ± 9.2**	72.7 ± 7.2**	59.1 ± 4.9**	58.5 ± 8.3**	25.9 ± 10.2	27 ± 4.4	5.0 ± 0.8	3.5 ± 0.4	0.14 ± 0.05	0.08 ± 0.04
E	76.5 ± 22.8*	79.2 ± 13.7*	81.9 ± 17.6	93.0 ± 14.4	4.1 ± 1.6*	5.4 ± 1.7	73.0 ± 11.7	78.1 ± 15.4	58.8 ± 4.2	61.3 ± 6.1*	30.2 ± 12.6	25.4 ± 10.0	5.2 ± 1.2^¥^	3.4 ± 0.33^¥^	0.14 ± 0.06	0.09 ± 0.05

^¤^The number of animals for the *C*_*p*_ and *C*_met_ was *N* = 5 due to a technical problem with the GCMS analysis of the plasma samples of two animals.

Significantly different from the previous measurement: *P < 0.05, **P < 0.01 level. ^¥^Significant differences between groups at the *P* < 0.05 level.

**Table 3:** Suppression ratio (SR: %), total power (TP_EEG_: *μ*V), spectral edge frequency (SEF: Hz), bispectral index (BIS), approximate entropy (AE), permutation entropy (PE), SR corrected SEF (SRSEF: Hz), and SR corrected PE (SRPE) at each study phase: A: right before the start of bleeding; B: in the end of bleeding/start of the waiting period; C: in the end of the waiting period/start of the volume replacement period); D: in the end of the volume replacement period/start of the final phase; and E: in the end of the study. G_LR_: group that received lactated Ringer's for volume replacement; G_HES_: group that received hydroxyethyl starch for volume replacement. Mean ± standard deviation is shown. The number of animals (*N*) in each group for each parameter is shown.

	SR (%)		TP_EEG_ (*μ*V)		SEF (Hz)		BIS		AE		PE		SRSEF (Hz)		SRPE	
	G_LR_ (*N* = 6)	G_HES_ (*N* = 6)	G_LR_ (*N* = 6)	G_HES_ (*N* = 6)	G_LR_ (*N* = 6)	G_HES_ (*N* = 6)	G_LR_ (*N* = 6)	G_HES_ (*N* = 6)	G_LR_ (*N* = 6)	G_HES_ (*N* = 6)	G_LR_ (*N* = 6)	G_HES_ (*N* = 6)	G_LR_ (*N* = 6)	G_HES_ (*N* = 6)	G_LR_ (*N* = 6)	G_HES_ (*N* = 6)
A	0.0 ± 0.0	5.9 ± 14.4	59.4 ± 3.3	59.6 ± 5.3	14.9 ± 0.9	14.9 ± 1.3	64.2 ± 5.7	55.8 ± 7.7	0.74 ± 0.15	0.76 ± 0.12	0.86 ± 0.09	0.84 ± 0.09	14.9 ± 0.9	13.9 ± 1.3	0.86 ± 0.09	0.79 ± 0.16
B	0.23 ± 0.56	7.1 ± 16.9	57.7 ± 3.9**	57.9 ± 5.2**	14.7 ± 1.5	15.4 ± 1.9	56.3 ± 7.4*	56.0 ± 15.9*	0.82 ± 0.13	0.77 ± 0.13	0.87 ± 0.09	0.85 ± 0.10	14.7 ± 1.6	14.1 ± 1.7	0.87 ± 0.09	0.79 ± 0.18
C	2.5 ± 5.1	11.9 ± 29.1	56.3 ± 4.6**	57.5 ± 5.2**	15.6 ± 1.3	15.6 ± 0.8	59.6 ± 5.8	55.1 ± 21.4	0.84 ± 0.12	0.76 ± 0.15	0.89 ± 0.09*	0.85 ± 0.09*	15.1 ± 0.9	13.8 ± 4.5	0.87 ± 0.10	0.74 ± 0.25
D	0.15 ± 0.34	13.6 ± 33.3	57.7 ± 3.3**	57.9 ± 6.7**	14.9 ± 0.6	15.1 ± 1.5	63.3 ± 4.4	51.9 ± 22.1	0.83 ± 0.12	0.78 ± 0.12	0.85 ± 0.09*	0.84 ± 0.09*	14.9 ± 0.5	12.2 ± 4.4	0.85 ± 0.09	0.72 ± 0.29
E	0.0 ± 0.0	9.7 ± 23.7	57.8 ± 3.2	58.3 ± 5.8	15.6 ± 1.2	15.0 ± 1.2	63.1 ± 3.5	53.2 ± 18.0	0.82 ± 0.14	0.77 ± 0.15	0.86 ± 0.09	0.84 ± 0.09	15.6 ± 1.2	13.3 ± 3.1	0.86 ± 0.09	0.76 ± 0.22

Significantly different from the previous measurement: *P < 0.05, **P < 0.01 level.
